# Supplementary material for: Determinants of breastfeeding self-efficacy among postpartum women in rural China: A cross-sectional study
Source: PLoS One. 2022 Apr 7;17(4):e0266273. doi: 10.1371/journal.pone.0266273 (PMC8989199; doi:10.1371/journal.pone.0266273)
Supplement: S1 Table — (DOCX) [file pone.0266273.s001.docx]

|  | |
| --- | --- |
| Did the baby’s mom have any of the following concerns or difficulties in the first 2 weeks of breastfeeding? Ask Q1-19 one by one. | |
| Questions | Answers |
| 1. Breast pains. | 1=Yes 2=No 999=Don’t know |
| 2. Back pains. |  |
| 3. Baby had trouble sucking or latching on onto the breast. |  |
| 4. Sore, cracked, or bleeding nipples. |  |
| 5. Not producing enough parent’s milk. |  |
| 6. C-Section affected breastfeeding. |  |
| 7. Episiotomy (cut vagina). |  |
| 8. Doctor suggested not to breastfeed. |  |
| 9. Baby choked when breastfeeding. |  |
| 10. Baby wouldn’t wake up to nurse regularly enough. |  |
| 11. Baby was not interested in nursing or got distracted. |  |
| 12. Baby nursed too often. |  |
| 13. Parent’s milk taking too long to secrete. |  |
| 14. Baby didn’t gain enough weight or lost too much weight. |  |
| 15. Not enough time to feed child. |  |
| 16. Infection of the breasts (e.g., abscess, yeast). |  |
| 17. Clogged milk duct. |  |
| 18. Breast engorgement. |  |
| 19. Parent’s milk leaked too much. |  |
