# Supplementary material for: Determinants of breastfeeding self-efficacy among postpartum women in rural China: A cross-sectional study
Source: PLoS One. 2022 Apr 7;17(4):e0266273. doi: 10.1371/journal.pone.0266273 (PMC8989199; doi:10.1371/journal.pone.0266273)
Supplement: S2 Table — (DOCX) [file pone.0266273.s002.docx]

|  | |
| --- | --- |
| **Questions** | **Answers** |
| 1.The first milk after birth is bad for your baby and should be discarded. Is this correct? | 1=Correct |
|  | 0=Wrong |
|  | 999=Don't know |
| 2.Women with small breasts cannot produce enough milk to feed a baby. Is this correct? | 1=Correct |
|  | 0=Wrong |
|  | 999=Don't know |
| 3.It is important to feed your baby water in addition to breastmilk. Is this correct? | 1=Correct |
|  | 0=Wrong |
|  | 999=Don't know |
| 4.Breastfeeding mothers should drink more than usual in order to ensure a good milk supply. Is this correct? | 1=Correct |
|  | 0=Wrong |
|  | 999=Don't know |
| 5.When should you breastfeed your child? | 1=Right before you have a meal |
|  | 2=Right after you finish a meal |
|  | 3=Whenever he/she is hungry |
|  | 4=Every three hours, no matter what |
|  | 999=Don't know |
| 6. When should you start to introduce water to your child? | 1=Immediately |
|  | 2=Within the first month |
|  | 3=When he starts to eat solid foods |
|  | 4=When he starts to crawl |
|  | 999=Don't know |
| 7. When should you start to introduce formula to your child? | 1=When he is around 3 months old |
|  | 2=When he is around 6 months old |
|  | 3=When he is around 1 year old |
|  | 4=Most babies don’t need to ever drink formula |
|  | 999=Don't know |
| 8.When should you start to introduce soft or semi-solid foods to your child? | 1=Right away |
|  | 2=When he is around 6 months old |
|  | 3=When he is around 1 year old |
|  | 4=When he is around 18 months old |
|  | 999=Don't know |
| 9.Which of the following foods is the best source of iron? | 1=Pork |
|  | 2=Egg |
|  | 3=Porridge |
|  | 4=Sweet potato |
|  | 999=Don't know |
| 10.What is the best way to know if your child has anemia? | 1=He looks smaller or skinnier than other babies his age |
|  | 2=His hair looks lighter in color |
|  | 3=Blood test from doctor |
|  | 4=He has trouble breathing |
|  | 999=Don't know |
| 11.If you are feeling sad or overwhelmed, which of the following is a good strategy for coping? | 1=Keep it to yourself, everyone has problems |
|  | 2=Ask for help from friends and family |
|  | 3=Eat more |
|  | 4=Spend more time on WeChat Moments |
|  | 999=Don't know |
| 12.What is the best way to prevent your baby from getting a cold? | 1=Wash hands regularly |
|  | 2=Limit trips outside of the house |
|  | 3=Feed him formula instead of breastmilk |
|  | 4=Always have him wear a hat |
|  | 999=Don't know |
