# Supplementary material for: Determinants of breastfeeding self-efficacy among postpartum women in rural China: A cross-sectional study
Source: PLoS One. 2022 Apr 7;17(4):e0266273. doi: 10.1371/journal.pone.0266273 (PMC8989199; doi:10.1371/journal.pone.0266273)
Supplement: Attachment — Submitted filename: Predictors of breastfeeding self Comment.docx [file pone.0266273.s004.docx]

**Predictors of breastfeeding self-efficacy among postpartum women in rural China: A cross-sectional study**

Dear Authors,

Thank you for the chance to review the manuscript. My detailed remarks on the review can be found here.

1. In the title should be used an appropriate word/term in line with epidemiological study deign, which is predictors leads to case-control rather than cross-sectional study. Please modify it?
2. In the abstract section line 37-39, all of the factors inversely associated with the predictors of BSE, but we are interpreting with a wrong ways. Please see and correcte accordingly.
3. Your conclusion should be consider drawing out the “so whats” of your findings to drive the points, but we are stated your conclusion that are the roles of health professionals.
4. Introduction; Your manuscript missing a section which generally describes the breastfeeding self-efficacy among postpartum women landscapes in China. What is breastfeeding self-efficacy? Why suggests breastfeeding self-efficacy among postpartum women? Has breastfeeding self-efficacy helped elsewhere? What is the general breastfeeding self-efficacy rate? All these you can touch on briefly?
5. Method; line 123-124: As stated above when use the term ‘predictors’ the design should be case-control.
6. Sample; line 139: why we use survey for this particular study, and also how to selected the actual participant of the study. Please elaborate it?
7. Line 141-150: These sentences are unclear. Kindly revisit.
8. Line 181: I am not sure you can use the word “extensive” as your manuscript has not really demonstrated the “extensiveness” of your approach.
9. Line 268; ‘November to December, 2019’ are you sure this was an enough time to conducted enumeration based on your study area coverage? Please see again and modify accordingly.
10. Line 273; why we used only 20 participants as pilot study and what was your reference’s we used only 20 participants?
11. Age category; which age classification are used in the study, which was ’18-30 Vs >31’?
12. Line 305-306 and line 315-316; we used mean score ± standard deviation, sure that the data distribution is normal?
13. Discussion: your discussion could be further developed so it doesn’t appear like a repetition of your objectives.
14. Line 464-466; this is the limitation of the study design itself not yours, please modify it. Why we are incorporating your strength?
15. General comment; There are several grammatical issues in manuscript which make it difficult for the readers to grasp the important points to convey in sections where they occur. I suggest you critically review the manuscript or possibly the service of copyeditor.

**Thanks!!!**
